# Supplementary material for: Exosome-Based Cell Homing and Angiogenic Differentiation for Dental Pulp Regeneration
Source: Int J Mol Sci. 2022 Dec 27;24(1):466. doi: 10.3390/ijms24010466 (PMC9820194; doi:10.3390/ijms24010466)
Supplement: Supplementary file 1 [file ijms-24-00466-s001.zip › ijms-2041345-supplementary.pdf]

Supplementary Table S1. List of novel miRNAs in Exo-G.

miRDeep 2 score	Consensus Mature sequence	Total read counts	Chromosome	Start	Stop	Strand
3379.5	GCTGTGATGGCCGAGTGG	6656	11	30795998	30796065	+
1604.3	GAAGTCGCAGCTCTCTGGGC	3143	14	163848014	163848071	-
1017.7	GGGGATGTAGCTCAGTGG	2002	11	31944616	31944678	+
694.5	TCAAAAAGTCGGCGGGAAATGC	1359	GL018776	1023377	1023447	-
590.5	AGCCAGCTCTGTGGCGCAGTGG	1164	7	34972538	34972589	+
350	TTCCTGTGCTGTTCTCGGTAG	684	GL018700	9256430	9256486	-
350	TTCCTGTGCTGTTCTCGGTAG	684	GL018700	9286959	9287015	-
344	TTCCTGTGCTGTTCTCGGTAG	672	GL018700	9253158	9253214	-
343.9	TTCCTGTGCTGTTCTCGGTAG	672	GL018700	9246701	9246757	-
343.9	TTCCTGTGCTGTTCTCGGTAG	672	GL018700	9272391	9272447	-
242.1	TGGGAACCTTGGGCAGGAAGT	471	GL018789	1006726	1006784	-
198.6	GTGACCTGTGGTGCTGGTGG	394	GL018724	1521599	1521649	-
196.9	TACTCAGAGCGGGACTGGAATGT	383	GL018700	9301745	9301801	-
196.8	TACTCAGAGCGGGACTGGAATGT	383	GL018700	9297778	9297834	-
176.4	TTGGCTGAGACAGTGTGACAT	343	12	148107814	148107872	+
174.9	TACCTAGAACAGCAACGGAGTGT	341	GL018700	9249628	9249684	-
150	TACCTAGAACAGCAACGGAGTGT	292	GL018700	9259730	9259786	-
135.4	ACCGCGTCTCCGCTCCCCGCAG	269	15	76135699	76135759	-
127.7	TTCTGGGCAGGCATCGCGCTGG	254	GL018928	57136	57193	+
125.4	AGGGGCTGGCACTGTGGCAT	252	GL018822	301512	301571	+
108.6	TGTCTCCTGTGCACTTCCCAGG	219	17	81223265	81223340	-
72.3	TGCCTGTGCCCCTTGGGAGTCT	146	AAGW02083711	3744	3803	-
72.3	TGCCTGTGCCCCTTGGGAGTCT	146	AAGW02083842	3863	3922	-
72.3	TGCCTGTGCCCCTTGGGAGTCT	146	GL019111	106599	106658	-
72.3	TGCCTGTGCCCCTTGGGAGTCT	146	GL019111	136410	136469	-
64.1	TGCAGGAACAAGCATGTGTAT	123	5	36025250	36025306	+
58	AGGGGCCAGTGCTGTGGTGTAG	110	7	13262868	13262944	+
57	TGTGGGGACCATTGTTGAGTGC	118	17	73354107	73354166	+
52.9	CCGTGACCGAGAGCGAGAACGA	101	19	43996541	43996610	+
47.4	TTCCTGTGCTGTTCTGGGTAG	90	GL018700	9242143	9242199	-
47.1	TGACTGCAGTGCCAGTGTCCCAT	90	14	153930371	153930428	+
46.3	TTCTGCCTGTGTAAGGAATGC	99	17	11866993	11867054	+
46	GAAGCGTAGGCGTGCTGGTGG	94	AAGW02083711	3160	3217	-
45.5	GAAGCGTAGGCGTGCTGGTGG	93	AAGW02083842	3276	3332	-
45.5	GAAGCGTAGGCGTGCTGGTGG	93	GL019111	106017	106073	-
39	ATGATTTTCAGCTAAGAATGG	74	7	99353953	99354012	-
37.4	TTCCTGGAACGCTCGGGGAGC	70	GL018725	923382	923437	-
37.1	AGGGGCTGGCACTGTGGCAC	80	GL019312	53901	53961	-
35.8	TGGAACCAGGACCTGCGGGGGC	66	AAGW02082842	671	727	-
32.7	TTGGGACTGGTGCTGCGACG	61	2	83442936	83442996	-
32.4	AGGGACTGGCTCTGTGGCG	69	12	87153697	87153757	-
30.6	TGGCCTGCAGTGCTGGCATCCT	58	19	31771015	31771069	+
29.6	TAAGGGCTGGCGATGTGGCAT	55	GL018717	1575634	1575696	+

26.6	TCACCACCAAGGAACCAACCAC	48	4	35492486	35492547	+
24.5	TACCTAGAACAGCAATGGAGTGT	49	GL018700	9263075	9263130	-
22.5	GAAGGTGGCCTGCCCTGTTCT	41	GL019134	104104	104165	-
21.3	ACACAACATTGAAGGAGGAAT	52	19	25357266	25357324	+
21	CACCTGCAGTGCCGGCATCCCAT	40	17	49336057	49336114	+
20.4	TTGGGGCTGGCACTGTGGCAT	38	17	8009414	8009472	+
17.9	CGCAGGTCTGGGGTCTGAGCTGC	32	GL019162	55775	55837	-
17	TGCAGTATGGGCCCCGTCCTG	30	GL019015	38527	38585	+
16.7	CCACCTGCAGTGCTGGCATCCC	30	9	36322677	36322732	+
16.5	TACTCAGAGAGGCACTGGCATGT	29	GL018700	9301076	9301132	-
16.2	ACGGTCCTCTGCCCACTCAG	36	9	99528136	99528196	+
15.4	TGGGAGACAGCAGAGAGAGGCT	28	2	130747420	130747482	+
15	AGGGGTGAGCCCCACAGAGTCT	35	12	20787416	20787486	-
14.3	CTTGCCTTTGCACTTGGGGGTT	24	X	108854306	108854365	-
13.3	TTGACCGAGGATCTGGCCCCAG	23	13	138301007	138301069	+
12.5	TTCTAGCAGTGCCGGCACCCCAT	22	11	65211194	65211252	+
12.5	GCGGAGCAGGTCTTGGATCAT	21	19	19424456	19424516	-
12.4	GATGGGAGTCAGTGGCCGTGG	21	21	12776073	12776128	+
12.3	TGAGAGTGGGTTCAAGTC	33	7	122613332	122613382	-
12.2	CAAGGCTGGGAGATGCTCTGA	21	10	10979171	10979231	-
12.2	CACCTGCAGTGCCGGCATCCCAT	31	12	45037872	45037930	-
12.1	TGCATCACATTGCCAGTCCTGA	21	10	16906372	16906438	-
11.3	CTTGGCTGTGGCTGCCCCAGGTTC	27	2	83422922	83422981	-
11.3	ACCACACGGGGCACCGCCGGGCTGG	26	GL018914	202622	202684	+
10.9	TGGGGCTGCCTGCCATGTGTGG	26	AAGW02083711	4853	4907	-
10.1	TTGAGGGCATCTTAAGAGAGGA	19	1	190847846	190847913	+
9.6	CAAAAGTTCATGGAAGTGGAAG	16	14	104692557	104692613	+
7.3	GGGCTGTATGTGTGCTTTTGC	13	1	70076462	70076522	-
7.2	GGGGCTGGCGCTGTGGTGCAG	11	7	166406071	166406125	-
7.2	AGGGCCGGCACTGTAGTATAGT	11	X	47659871	47659926	+
7.2	AGACAGGAAGGGTGTGTCTGT	11	GL019171	65160	65234	-
6.9	CAATCAGCCTACTGGGAACAT	26	1	123190047	123190103	+
6.8	TCTGTCTGCCCTCCGCTTGCT	10	9	7084950	7085008	-
6.7	ACCTGCATGTTAGTCTAATGTTCTG	11	4	77890296	77890385	+
6.3	CCCACCCACCCCTGTGAGATCG	8	GL018940	47229	47287	-
5.3	TGACCCCCGGCCGGACCCCAC	6	4	84827251	84827317	-
5.2	AAGGGATCGGCATTCTGGCAT	9	1	184713261	184713319	-
5.2	AGTCCAGGGCTTAGTCAGCGG	7	15	50955589	50955648	+
5	TCGTATCCACCTCTGTTGCAG	19	GL018831	47863	47943	+
5	ATCATGTATGATACTGCACACA	14	GL019074	61059	61121	+
4.5	TATTCAAGCCCAGCGGAGGTAC	5	2	159928226	159928285	+
4.5	TATGTTTGCTGTTTTGTGAGAC	10	7	172929928	172929992	-
4.4	CCCCCAAGGACACTGCCTGCCTAC	5	10	45287368	45287427	+
4.4	TAGGGAGAGAATGAGTGGACTGA	8	20	30157180	30157238	+
4.2	GTCGCACGCACGCCCGCCTCGG	12	1	130454171	130454237	-

Supplementary Table S2. List of novel miRNAs in Exo-A.

miRDeep 2 score	Consensus Mature sequence	Total read counts	Chromosome	Start	Stop	Strand
64482	GGTGCTGTAGGCTTTAAA	126510	19	54756247	54756283	+
486.7	GAAGTCGCAGCTCTCTGGGC	951	14	163848014	163848071	-
282.1	GTCTGAGCGGCGGCCACT	559	13	110485608	110485664	+
251.8	TGTCTCCTGTGCACTTCCCAGG	500	17	81223265	81223340	-
220.8	TCAAAAAGTCGGCGGGAAATGC	430	GL018776	1023380	1023447	-
70.2	CCGTGACCGAGAGCGAGAACGA	135	19	43996541	43996610	+
62.9	TTCCTGTGCTGTTCTCGGTAG	121	GL018700	9256430	9256486	-
62.9	TTCCTGTGCTGTTCTCGGTAG	121	GL018700	9286959	9287015	-
47.7	TCAAAGGAAGAAGACTGGCGT	91	11	3609112	3609171	+
44.9	TTGGCTGAGACAGTGTGACAT	85	12	148107814	148107871	+
25.8	CAATCAGCCTACTGGGAACAT	63	1	123190047	123190103	+
20.6	AGGGGTGAGCCCCACAGAGTCT	46	12	20787417	20787486	-
17.5	TGGGCCCACAGAGACTCTGG	31	GL018897	256545	256602	-
17.4	TTCCTGTGCTGCTCTGAGTAG	40	GL018700	9249628	9249684	-
17.3	CCTAGAGTGTCGGCATCCCTTT	40	GL018822	301512	301571	+
16.8	TTCTGCCTGTGTAAGGAATGC	41	17	11866993	11867054	+
15.7	ATCATGTATGATACTGCACACA	35	GL019074	61059	61121	+
13.7	TGTGGGGACCATTGTTGAGTGC	33	17	73354107	73354166	+
13.3	CGCAGGTCTGGGGTCTGAGCTGC	23	GL019162	55776	55837	-
13	ATGATTTTCAGCTAAGAATGG	23	7	99353950	99354012	-
11.6	GTGTATGATGACTGGATT	20	GL018725	3255292	3255368	+
10.9	GCGGAGCAGGTCTTGGATCAT	18	19	19424457	19424516	-
10.8	TACTCAGAGCGGGACTGGAATGT	18	GL018700	9301745	9301801	-
10.7	TACTCAGAGCGGGACTGGAATGT	18	GL018700	9297778	9297834	-
9.9	CATCCAGAAGTCCAGGGACTTT	20	GL018705	625149	625204	+
9.8	TTCCTGGAACGCTCGGGGAGC	16	GL018725	923381	923437	-
8.8	TACCTAGAACAGCAACGGAGTGT	15	GL018700	9259730	9259786	-
6.9	TGAACCTGTGATGCCAGCATCC	10	8	33724831	33724887	-
6	TATGTTTGCTGTTTTGTGAGAC	13	7	172929928	172929992	-
6	CTTCCTGGCTGACGTGGAGTCC	8	20	9171823	9171886	+
5.7	GGCAGGGCCTGCTTCCTTCC	8	GL019134	104104	104165	-
5.2	CGTCTGGGCTGGCCCGAGCG	14	GL019068	92002	92058	-
5.1	TACCTAGAACAGCAATGGAGTGT	7	GL018700	9242142	9242199	-
5	CCTCTGCACTCTCTCTCACCAG	14	GL018821	265484	265554	-
4.4	TCTGGCTTCAAGGTGCTGTGT	5	GL018725	2252226	2252285	-
4.1	ACACAGCAGACTCAGAGG	9	14	122355007	122355074	+
